# Oxidative Stress Markers and Heat Shock Proteins in Non-Obese Women with Polycystic Ovary Syndrome Are Not Elevated and Show No Correlation with Vitamin D

**DOI:** 10.3390/biomedicines11072044

**Published:** 2023-07-20

**Authors:** Manjula Nandakumar, Thozhukat Sathyapalan, Alexandra E. Butler, Stephen L. Atkin

**Affiliations:** 1 Research Department, Royal College of Surgeons in Ireland Bahrain, Busaiteen, Adliya 15503, Bahrain; mnandakumar@rcsi.com (M.N.); satkin@rcsi.com (S.L.A.); 2 Academic Endocrinology, Diabetes and Metabolism, Hull York Medical School, Hull HU6 7RU, UK; thozhukat.sathyapalan@hyms.ac.uk

**Keywords:** polycystic ovary syndrome, oxidative stress, heat shock proteins, vitamin D_3_

## Abstract

Introduction. Oxidative stress (OS) is recognized in the pathophysiology of polycystic ovary syndrome (PCOS). OS results in intracellular reactive oxygen species generation, causing oxidative protein damage that is protected by heat shock proteins (HSPs). Vitamin D is thought to reduce and protect against OS; therefore, OS, HSP, and vitamin D levels may be associated with PCOS. However, their expression in PCOS without underlying inflammation is unknown. Methods. In this exploratory study, the plasma levels of 7 OS proteins and 10 HSPs that are affected by the OS process were measured using Slow Off-rate Modified Aptamer (SOMA)-scan plasma protein measurements in non-obese, non-insulin resistant women with PCOS (*n* = 24) without systemic inflammation and control (*n* = 24) women; the cohorts were matched for weight and age. The OS proteins and HSPs were correlated with 25-hydroxy vitamin D_3_ (25(OH)D_3_) and the active form, 1,25-dihydroxyvitamin D_3_ (1,25(OH)_2_D_3_), as measured by isotope-dilution liquid chromatography tandem mass spectrometry. Results. The PCOS women versus the controls had comparable insulin resistance and systemic inflammation (C-reactive protein 2.0 mg/L vs. 2.3 mg/L, *p* > 0.05), but higher free androgen index and anti-mullerian hormone levels. Among the OS proteins, only esterase D (ESD; *p* < 0.01) was elevated in PCOS and the HSPs did not differ between the PCOS and control women. There was no correlation of 25(OH)D_3_ or 1,25(OH)_2_D_3_ with any of the proteins. Conclusions. In a PCOS population that was non-obese and without insulin resistance and systemic inflammation, only ESD was elevated in PCOS, whilst the other OS proteins and HSPs were not elevated. Further, none of the OS proteins or HSPs were correlated with either 25(OH)D_3_ or 1,25(OH)_2_D_3_ in either cohort of women or when both cohorts were combined, indicating that the OS and HSP responses were largely absent and not affected by vitamin D in a non-obese PCOS population.

## 1. Introduction

Affecting 5–10% of women, polycystic ovary syndrome (PCOS) is the most prevalent endocrine abnormality in premenopausal women [1]. PCOS is a diagnosis of exclusion that can be determined by recognized international criteria [2] and it is estimated that 70% of women with PCOS remain undiagnosed [3]. It is well recognized that polycystic ovary syndrome (PCOS) is a metabolic disease with an increased prevalence of type 2 diabetes, hypertension, dyslipidemia, and enhanced cardiovascular risk factors in affected women [4]. The pathophysiological mechanisms underlying these features found in PCOS are still unclear, though oxidative stress (OS), perhaps primarily due to obesity, has been implicated [5,6]; however, other mechanisms involving OS mediated through hyperandrogenemia, vitamin D levels, and insulin resistance have also been reported [7,8], together with other cellular biomarkers such as the dysregulation of heat shock proteins [9], coagulation markers [10], and the complement pathway proteins, through a combination of obesity and insulin resistance as the underlying mediators [11,12,13,14].

Whilst obesity is common in PCOS, it is likely not a primary cause, as is reflected in the high prevalence of PCOS among non-obese populations [15]. However, obesity contributes to the exacerbation of its underlying pathophysiology, especially cardiovascular risk factors such as insulin resistance, glucose intolerance, and dyslipidemia. Weight loss decreases these cardiovascular risk markers, as is particularly noted following bariatric surgery [16]; however, no medical intervention has been shown to decrease cardiovascular events or delay the onset of diabetes [17]. Chronic systemic inflammation is seen in obesity, which is reflected in the enhanced serum levels of inflammatory cytokines and lymphocyte function changes [18,19,20]. High-sensitivity C-reactive protein (hs-CRP) is a measure of inflammation and women with PCOS have significantly increased hs-CRP concentrations [21]. It has been recognized that CRP elevation is independently related to insulin resistance and may be related to coronary heart disease and cardiovascular events in PCOS [22]. CRP has been shown to be a strong predictor of cardiovascular events in women [23].

PCOS is associated with insulin resistance, an increased risk of diabetes, non-alcoholic fatty liver disease (NAFLD), and increased cardiovascular (CV) risk, independent of obesity [24,25]. Insulin resistance results from a defect in insulin action, including insulin-mediated glucose transport and its signaling pathway [26]. Hyperinsulinemia results in an increased risk of type 2 diabetes in PCOS [27]. In addition, hyperinsulinemia leads to dyslipidaemia, which is seen as a common feature in PCOS and contributes to its documented cardiometabolic abnormalities [28].

**Figure 1:**
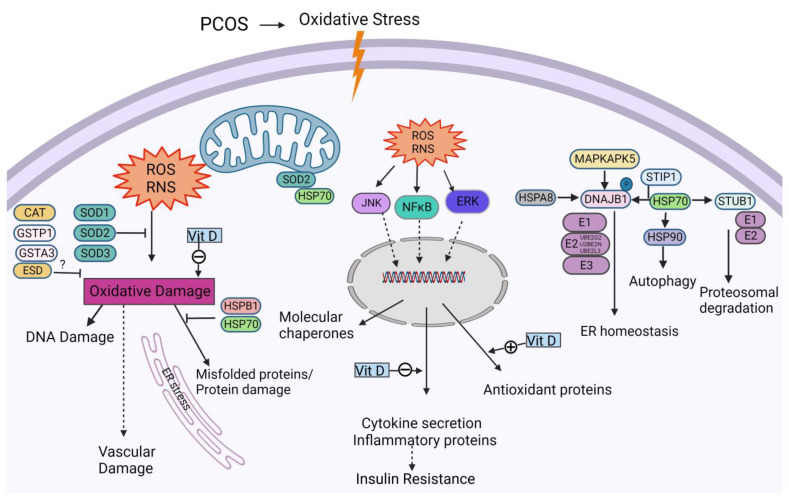
Schematic illustration to show the interactions of vitamin D, heat shock proteins, and oxidative-stress-related proteins. In PCOS-associated oxidative stress, the ROS/RNS produced are linked to several pathways (examples being the NFkB, JNK, and ERK signaling pathways) which are involved in regulating pro-oxidant genes which lead to oxidative damage and antioxidant genes which protect from oxidative damage. Oxidative stress results in protein misfolding and oxidative damage of proteins, thereby causing ER stress. Heat shock proteins work to reduce the ER stress and maintain ER homeostasis. Many proteins which play a role as antioxidants are produced during oxidative stress which, by themselves or together with HSPs, help in reducing oxidative damage. Vitamin D is reported to reduce oxidative-stress-related proteins such as inflammatory proteins/secreted cytokines and to upregulate antioxidant enzymes/proteins. HSP90 alpha (HSP90AA1, HSP90AB1, HSP90 beta, and HSP90 dimer); HSPA8, heat shock cognate 71 kDa protein; DNAJB1, DnaJ homolog subfamily B member 1; MAPKAPK5, MAP kinase-activated protein kinase 5; STIP1, stress-induced phosphoprotein 1; E1, ubiquitin-activating enzyme; E2, ubiquitin-conjugating enzyme 2; UBE2L3, ubiquitin-conjugating enzyme E2 L3; UBE2N, ubiquitin-conjugating enzyme E2 N; E3, ubiquitin ligases; STUB1, STIP1 Homology And U-Box Containing Protein 1; SOD1, Superoxide dismutase1; SOD2, Superoxide dismutase2; SOD3, Superoxide dismutase3; CAT, Catalase; ESD, Esterase D; GSTP1, Glutathione S-transferase P1; GSTA3, Glutathione S-transferase A3; JNK, c-Jun N-terminal kinases; NFkB, Nuclear factor-kappa B; ERK, extracellular signal-regulated kinases; Vit D, Vitamin D; HSP90 alpha (HSP90AA1, HSP90AB1, HSP90 beta, HSP90 dimer); HSPA8, heat shock cognate 71 kDa protein; DNAJB1, DnaJ homolog subfamily B member 1; MAPKAPK5, MAP kinase-activated protein kinase 5; STIP1, stress-induced phosphoprotein 1; E1, ubiquitin-activating enzyme; E2, ubiquitin-conjugating enzyme 2; UBE2L3, ubiquitin-conjugating enzyme E2 L3; UBE2N, ubiquitin-conjugating enzyme E2 N; E3, ubiquitin ligases; STUB1, STIP1 Homology And U-Box Containing Protein 1; SOD1, Superoxide dismutase1; SOD2, Superoxide dismutase2; SOD3, Superoxide dismutase3; CAT, Catalase; ESD, Esterase D; GSTP1, Glutathione S-transferase P1; and GSTA3, Glutathione S-transferase A3.

Increased OS is caused by an imbalance between oxidants and antioxidants that then results in the formation of intracellular reactive oxygen species (ROS). The circulating markers of OS have been reported by some as being abnormal in PCOS [29], though these reports have been conflicting [5]. The generation of ROS leads to protein oxidation that may affect many biological parameters, ultimately resulting, for example, in vascular injury [30]. However, OS protein damage is limited by the protective HSP response [31]. HSPs function as molecular chaperones that prevent protein misfolding in order to prevent intracellular dysregulation [32], and they are constitutively expressed at low levels but become elevated with a stress stimulus such as OS [33] (Figure 1). Impaired proteins that are misfolded could result in cellular dysregulation and are channeled by HSPs to the ubiquitin proteasome system (UPS) to effect their degradation [34]; following ubiquination, proteolysis occurs via the 26S proteasome [35]. HSPs can be seen to be involved in the regulation and functional facilitation of the critical enzymes in inflammation, apoptosis, metabolism, and cell signaling [36,37]. HSPs are becoming increasingly recognized as having an important role in the pathophysiology of PCOS [9], acting as protection against PCOS-associated increased OS, inflammation, and insulin resistance [9].

Vitamin D deficiency is highly prevalent in women with PCOS, with 67–85% exhibiting a severe deficiency. Vitamin D deficiency is associated with the PCOS pathognomonic features of obesity, increased insulin resistance, and elevated levels of testosterone [38,39]; however, whilst vitamin D deficiency may not exacerbate these features in the setting of PCOS [40], vitamin D replacement has been suggested to benefit both insulin resistance and steroidogenesis in obese women with PCOS [41,42]. The vitamin D deficiency reported to be associated with insulin resistance is considered to be obesity dependent [43] and, indeed, vitamin D deficiency increases with obesity as the vitamin D is sequestered in adipose tissue. Vitamin D has been suggested to be a key controller of systemic inflammation, oxidative stress, and mitochondrial function and is a potent antioxidant in its own right [44] Vitamin D_3_ (cholecalciferol) is produced endogenously in the skin as a consequence of ultraviolet B radiation (UV-B), acting on 7-dehydrocholesterol that is hydroxylated at position 25 to 25-hydroxy vitamin D_3_ (25(OH)D_3_). 25(OH)D_3_ is transported to the kidney, where 1-alpha hydroxylase converts it into the active form, 1,25(OH)_2_D_3_ [45].

Obesity, inflammation, and insulin resistance, with accompanying OS, are highly correlated with PCOS; thus, statistical regression adjustment for BMI/insulin resistance/inflammation is misleading, as it will over-adjust the effects of PCOS. Therefore, this study was performed on a cohort of women with PCOS who were non-obese versus control women; the cohorts were matched for BMI, insulin resistance, and systemic inflammation to determine the association of circulatory OS proteins and HSPs with PCOS and whether there was evidence of modulation by vitamin D metabolites.

## 2. Materials and Methods

The approval for this study was granted by The Yorkshire and The Humber NRES ethical committee, UK (February 2003; study approval number 02/03/043).

Seven plasma OS protein levels and ten plasma HSP levels were determined in 24 women with PCOS and 24 control women, all of whom were patients attending the Hull IVF clinic [45]. The ages and body mass indexes (BMI) of the control women were matched to those of the women with PCOS. For each of the two cohorts, the control and PCOS women, the demographic data are detailed are Table 1 [45]. The Rotterdam consensus criteria were used for diagnosis, following the exclusion of confounding conditions such as non-classical 21-hydroxylase deficiency, Cushing’s disease, hyperprolactinemia, or an androgen-secreting tumor. The specific Rotterdam criteria for the diagnosis of PCOS include oligomenorrhea or amenorrhea; biochemical hyperandrogenemia or clinical hyperandrogenemia with a Ferriman–Gallwey score of >8 plus a free androgen index of >4; and polycystic ovaries, as determined by transvaginal ultrasound [46]. The study subjects had no other conditions/illnesses and the study’s inclusion criteria required them to be medication-free (including over-the-counter antioxidants) for at least the 9 months prior to enrollment in the study. The procedures performed on the women in this study were aligned with the Helsinki declaration and its later amendments or comparable ethical standards.

Seasonal fluctuations in vitamin D metabolite levels are well recognized and may contribute to their variability. Nine minutes of daily sunlight exposure would be sufficient for vitamin D sufficiency in northern England; therefore, to ensure the targets were met and to reduce potential variability, the subjects were recruited from March to September [47]. Vitamin D supplement consumption was an exclusion criterion of the study.

Bloods drawn from the women in the fasting state were centrifuged at 3500× *g* for 15 min, then aliquoted and frozen (−80 °C) in preparation for analysis. The aliquots of blood were analyzed for the following: insulin, sex hormone binding globulin (SHBG) (DPC Immulite 200 analyser, Euro/DPC, Llanberis, UK), and plasma glucose (Synchron LX20 analyser, Beckman-Coulter, High Wycombe, UK). To calculate the free androgen index (FAI), the total testosterone was divided by the SHBG, and the result was multiplied by 100. To calculate the insulin resistance (IR), the homeostasis model assessment (HOMA-IR) was employed. To quantify the serum testosterone, isotope-dilution liquid chromatography tandem mass spectrometry (LC-MS/MS) methodology was utilized [45]. Likewise, to quantify the serum vitamin D levels, isotope-dilution liquid chromatography tandem mass spectrometry (LC-MS/MS) was utilized. Vitamin D metabolites (25(OH)D_3_ and 1,25(OH)_2_D_3_), together with labeled internal standards (d6-25(OH)D_3_ and d6-1,25(OH)_2_D_3_), were extracted simultaneously from 250 µL of serum with the use of supportive liquid–liquid extraction and Diels–Alder derivatization being undertaken prior to performing an LC-MS/MS analysis. To obtain chromatographic separations, the following was employed: a Hypersil Gold C18 column (150 × 2.1 mm; 1.9 µ) at a flow rate of 0.2 mL/min, set to operate in the Electrospray Ionisation (ESI) positive mode, and an analysis performed using the reaction monitoring (MRM) method. The limit of quantification (LOQ) was determined to be as follows: 0.5 ng/mL for 25(OH)D_3_ and 10 pg/mL for 1,25(OH)_2_D_3_, with a 25(OH)D_3_ cutoff of less than 20 ng/L (50 nmol/L) to define deficiency.

The circulatory levels of plasma proteins were measured using Slow Off-rate Modified Aptamer (SOMA)-scan plasma protein measurement technology (Somalogic, Boulder, CO, USA), as has been described in detail in prior reports [48]. Raw intensity normalization and hybridization, together with median signal and calibration signal determination, were based upon the referent standard samples that are incorporated into each plate, as has been previously detailed [49].

Measurements of the plasma levels of the following seven OS proteins were undertaken: Superoxide dismutase (SOD1); Glutathione S-transferase P (GSTP1); Glutathione S-transferase A3 (GSTA3); Esterase D (ESD); Catalase (CAT); Superoxide dismutase mitochondrial (SOD2); and Extracellular superoxide dismutase (SOD3). In addition, we measured 10 HSPs that are included in the Somascan panel: Heat shock protein HSP 90-alpha/beta (HSP90AA1/AB1); Heat shock cognate 71 kDa protein (HSP70; HSPA8); Heat shock protein beta-1 (HSP27, HSPB1); MAP kinase-activated protein kinase 5 (MAPKAPK5); DnaJ homolog subfamily B member 1 (DNAJB1, HSP40); Stress-induced-phosphoprotein 1 (STIP1); E3 ubiquitin-protein ligase CHIP (STUB1); Ubiquitin-conjugating enzyme E2 L3 (UBE2L3); Ubiquitin-conjugating enzyme E2 N (UBE2N); and Ubiquitin-conjugating enzyme E2 G2 (UBE2G2). As such, 7 OS-related proteins and 10 HSPs are presented herein.

### Statistics

Using nQuery version 9 (Statsol, Boston, MA, USA), a power analysis was performed on lipid peroxidation based upon prior reports on PCOS [50]; lipid peroxidation is the oxidative degradation of lipids to give a general measure of oxidative stress [50]. For 90% power and an alpha of 0.05 with a common standard deviation of 0.38, together with an effect size of 1.16, the required number of subjects was determined to be 17 per arm; however, this study was exploratory, as it was not possible to calculate the power for an individual OS or HSP protein, so 48 subjects were recruited to ensure adequate subject numbers. Both visual and statistical evaluations were performed on the data to assess normality. Independent t-tests were utilized when the data were normally distributed; alternatively, when the data were not normally distributed, as determined using the Kolmogorov–Smirnov test, the Mann–Whitney U non-parametric test was utilized. Bonferroni adjustment for multiple comparisons was undertaken. Correlation analyses between the vitamin D and the OS-related and HSP proteins were performed with Pearson coefficient. All the analyses were performed using Graphpad Prism version 10.0.0 (San Diego, CA, USA).

## 3. Results

### 3.1. Demographic Data

The non-obese PCOS cohort and the control women cohort were well matched for age, BMI, insulin resistance (HOMA), and inflammation (CRP), as these parameters did not differ between the two groups (Table 1). As expected, the PCOS cohort showed a classical elevation in the free androgen index and an elevated anti-Mullerian hormone. Neither 25(OH)D_3_ nor 1,25(OH)_2_D_3_ differed between the groups. Of the 48 women recruited, 28 were 25(OH)D_3_ deficient (<20 ng/mL (50 nmol/L) 25(OH)D_3_).

### 3.2. Oxidative Stress Results

The results of the circulatory OS proteins are shown in Table 2 for both the non-obese PCOS and control subjects. In these non-obese, non-insulin resistant PCOS subjects, only ESD was elevated (*p* < 0.01) compared to the controls, but there were no other differences in the other OS proteins following the Bonferroni correction for multiple comparisons.

As neither 25(OH)D_3_ nor 1,25(OH)_2_D_3_ differed between the groups, the groups were combined to increase the power of identifying an association between 25(OH)D_3_ or 1,25(OH)_2_D_3_ and the OS markers. These data are shown in Table 3 and Supplemental Figures S1 and S2 for OS with 25(OH)D_3_ and 1,25(OH)_2_D_3_, respectively. No associations between 25(OH)D_3_ or 1,25(OH)_2_D_3_ and any of the OS proteins were found.

When the patients were divided based on vitamin D deficiency, there were no differences in any of the OS proteins.

### 3.3. Heat Shock Protein Results

The results of the HSP proteins are shown in Table 2 for both the non-obese PCOS and control subjects. There were no differences in the HSPs following the Bonferroni correction for multiple comparisons in these non-obese, non-insulin resistant PCOS subjects.

As neither 25(OH)D_3_ nor 1,25(OH)_2_D_3_ differed between the groups, the groups were combined to increase the power of identifying an association between 25(OH)D_3_ or 1,25(OH)_2_D_3_ and the HSP markers. These data are shown in Table 3 and Supplemental Figures S3 and S4 for the HSPs with 25(OH)D_3_ and 1,25(OH)_2_D_3_, respectively. There were no associations between 25(OH)D_3_ or 1,25(OH)_2_D_3_ and any of the HSPs.

When the patients were divided based on vitamin D deficiency, there were no differences in any of the HSP proteins.

## 4. Discussion

These data show that, in non-obese PCOS subjects in which BMI systemic inflammation and insulin resistance were accounted for and did not differ to the controls, the OS proteins other than ESD and the HSPs were not elevated in PCOS. In addition, the OS proteins and the HSPs studied showed no correlation with either vitamin D, 25(OH)D_3_, or its active 1,25(OH)_2_D_3_.

Increased OS is recognized as being important in the pathogenesis and pathophysiological processes of PCOS [5,6]. Esterase D (ESD) is a nonspecific esterase that detoxifies formaldehyde. Several reports have detailed that increased ESD activity is associated with various physiological and pathological processes, including autophagy, but its signaling processes are poorly understood [51]. Whilst being recognized as being dysregulated in cancer and important in inhibiting cell growth [52], ESD measurement as an OS protein in PCOS has not been described before, and here it was significantly elevated, suggesting that its function in PCOS requires further investigation. However, as the other markers of OS were unaltered, this suggests that its elevation was reflecting another (as of yet unknown) process. Malondialdehyde is a measure of OS that is derived from lipid peroxidation, has been shown to be increased in several studies, and has, additionally, been suggested to be independent of obesity [53]; the study by Kuşçu and Var is the converse to what is shown here, though their study population was not non-obese. Glutathione transferase has been reported to be low in PCOS compared to controls [54], but increased in other reports [55], whilst in this study, it did not differ. SOD removes superoxide anions and has been shown to be increased in PCOS [56], though it was unaltered in this study. This suggests that when obesity, insulin resistance, and inflammation are accounted for, this population is at no greater risk than the control population for cardiovascular risk, and that hyperandrogenemia alone is not a contributory factor to the increased oxidative stress reported in PCOS.

No differences were seen in the HSPs in this population, which is perhaps unsurprising, as their regulation would be dependent on increased OS, systemic inflammation, insulin resistance, and obesity, which were not present in this study population. The finding of elevated ESD also suggests that it is not acting as an OS protein in this context, as there was no association with any of the HSPs.

In this cohort of non-obese, non-insulin resistant PCOS patients without systemic inflammation, our findings contrast with the reported changes in the OS and HSPs in PCOS and suggest that those reported changes are due to the underlying obesity, insulin resistance, and inflammation, rather than due to the underlying pathophysiology of PCOS. Oxidative stress is associated with both obesity and insulin resistance [57,58], which may act synergistically or in an additive fashion, as an increasing BMI results in increasing insulin resistance [59]. The cardiovascular risk factors of obesity, insulin resistance, and chronic inflammation are more linked to obesity rather than the pathophysiology of PCOS; however, teasing out what the contribution of the inherent PCOS pathophysiology is versus the relative effects of insulin resistance and obesity [15,60] is only possible with populations matched to exclude confounders, including ethnicity, that may affect PCOS [61]; the contributions of obesity, insulin resistance, and inflammation were specifically accounted for to circumvent these confounders.

No correlations were seen for either the 25(OH)D_3_ or the active 1,25(OH)_2_D_3_ levels with either the OS proteins or the HSPs. In addition, when the cohort was divided into vitamin D deficient and sufficient, there were no differences between the groups for either the OS or the HSPs in this cohort of non-obese individuals without insulin resistance or raised inflammatory markers. However, these data do not negate that the changes in the OS factors reported in obese PCOS are dependent on vitamin D levels or, conversely, that the deleterious effects of vitamin D deficiency are not mediated through the OS process in PCOS, as these may be affected by the obesity, inflammation, and insulin resistance that are commonly found in PCOS. A meta-analysis reported that vitamin D supplementation improved the OS parameters in PCOS [62], suggesting that vitamin D may be beneficial when OS is present. Whilst the kidneys produce 1,25-dihydroxy vitamin D for regulating the calcium and bone metabolism [63], it is recognized that 1,25(OH)_2_D can be produced externally to the kidneys though macrophage production [64]. Consequently, 1,25-dihydroxyvitamin D blood levels may not reflect the tissue level function that could be beneficial due to the local production of 1,25-dihydroxy vitamin D [65].

This study has a number of limitations. OS proteins were measured rather than the functional serum oxidative capacity, such as measuring malondialdehyde (MDA), 4-hydroxy-nonenal (HNE), and the F2-isoprostane 15(S)-8-iso-prostaglandin F2α (15(S)-8-iso-PGF2α). However, it is recognized that these methods are difficult and less precise due to analytical issues [66]. There are no studies that could have been utilized for a power analysis for this non-obese PCOS cohort; therefore, the potential differences were based on those of an obese PCOS population; therefore, a type 2 (beta) error cannot be excluded. All the study subjects were Caucasian, and therefore these results may not be generalizable to other ethnic populations. This study lays the foundation for dissecting the initiation of OS in PCOS, as we have shown here that OS in this population does not occur; the identification of PCOS cohorts with inflammation alone and insulin resistance alone without obesity would identify the causative mechanisms for OS in this population and those scenarios where vitamin D may afford protection.

In conclusion, in a PCOS population that was non-obese and without insulin resistance and systemic inflammation, only ESD was elevated in PCOS, whilst the other OS proteins and HSPs were not elevated. Further, none of the OS proteins or HSPs were correlated with either 25(OH)D_3_ or 1,25(OH)_2_D_3_ in either cohort of women, or when both cohorts were combined, indicating that the OS and HSP responses were largely absent and not affected by vitamin D in this non-obese PCOS population.

## Figures and Tables

**Table 1:** Demographics, baseline, hormonal, and metabolic parameters of the PCOS subjects and controls (mean ± SD). All parameters did not differ other than those marked ** = *p* < 0.01.

	Control (*n* = 24)	PCOS (*n* = 24)
Age (years)	32.5 ± 4.1	31 ± 6.4
BMI (kg/m^2^)	24.8 ± 1.1	25.9 ± 1.8
Fasting glucose (mmol/L)	4.9 ± 0.4	4.7 ± 0.8
HbA1C (mmol/mol)	30.9 ± 6.5	31.8 ± 3.0
HOMA-IR	1.8 ± 1.0	1.9 ± 1.6
SHBG (nmol/L)	104 ± 80	72 ± 62
Free androgen index (FAI)	1.3 ± 0.5	4.1 ± 2.9 **
CRP (mg L^−1^)	2.3 ± 2.3	2.7 ± 2.5
AMH (ng/mL)	24 ± 13	57 ± 14 **
25 hydroxy vitamin D_3_ (nmol/l)	46.2± 23.5	54.0 ± 27.4
1,25 Dihydroxy vitamin D_3_ (ng/mL)	0.03 ± 0.02	0.04 ± 0.2

BMI—Body Mass Index; HbA1c—glycated hemoglobin; HOMA-IR—Homeostasis model of assessment—insulin resistance; CRP—C reactive protein; SHBG—sex hormone binding globulin; and AMH—Anti-Müllerian hormone.

**Table 2:** Levels of circulatory heat shock and oxidative stress proteins in control women (*n* = 24) and women with polycystic ovary syndrome (PCOS) (*n* = 24). Levels are reported as mean ± 1 standard deviation of RFU (relative fluorescent units).

		Control	PCOS	*p*-Value
Heat shock proteins	HSP90AA1/AB1	6566 (11,881)	5160 (6109)	0.57
	HSP70 (HSPA8)	2140 (2361)	1606 (348)	0.23
	HSP27 (HSPB1)	2350 (1727)	3773 (4633)	0.13
	MAPKAPK5	985 (2314)	625 (504)	0.42
	DNAJB1 (HSP40)	447 (296)	485 (337)	0.66
	STIP1	7563 (7799)	6785 (4837)	0.65
	STUB1	1660 (3163)	1212 (1138)	0.48
	UBE2L3	1212 (635)	1322 (764)	0.55
	UBE2N	7696 (8053)	7014 (6284)	0.72
	UBE2G2	4642 (1853)	4401 (1002)	0.54
Oxidative stress proteins	SOD1	973 (621)	914 (325)	0.64
	GSTP1	3358 (2260)	3594 (763)	0.65
	GSTA3	209 (214)	182 (119)	0.53
	ESD	4606 (1887)	6184 (2632)	0.01
	CAT	22,445 (12,953)	22,749 (8629)	0.89
	SOD2	48,222 (10,590)	51,647 (12,763)	0.26
	SOD3	506 (455)	625 (710)	0.46

HSP90AA1/AB1: Heat shock protein HSP 90-alpha/beta; HSP70 (HSPA8): Heat shock cognate 71 kDa protein; HSP27 (HSPB1): Heat shock protein beta-1; MAPKAPK5: MAP kinase-activated protein kinase 5; DNAJB1 (HSP40): DnaJ homolog subfamily B member 1; STIP1: Stress-induced-phosphoprotein 1; STUB1: E3 ubiquitin-protein ligase CHIP; UBE2L3: Ubiquitin-conjugating enzyme E2 L3; UBE2N: Ubiquitin-conjugating enzyme E2 N; UBE2G2: Ubiquitin-conjugating enzyme E2 G2; SOD1: Superoxide dismutase; GSTP1: Glutathione S-transferase P; GSTA3: Glutathione S-transferase A3; ESD: Esterase D; CAT: Catalase; SOD2: Superoxide dismutase mitochondrial; and SOD3: Extracellular superoxide dismutase.

**Table 3:** Lack of correlation of circulatory heat shock and oxidative stress proteins with vitamin D_3_ (25(OH)D_3_) and its active form, 1,25(OH)_2_D_3_.

		25(OH)D_3_		1,25(OH)_2_D_3_	
		r-Value	*p*-Value	r-Value	*p*-Value
Heat shock proteins	HSP90AA1/AB1	0.2	0.17	0.02	0.9
	HSP70 (HSPA8)	0.06	0.67	0.02	0.93
	HSP27 (HSPB1)	0.22	0.14	0.09	0.59
	MAPKAPK5	0.08	0.6	0.26	0.12
	DNAJB1 (HSP40)	0.24	0.1	0.0005	0.98
	STIP1	0.06	0.69	0.03	0.87
	STUB1	0.05	0.73	0.06	0.73
	UBE2L3	0.0002	0.96	0.03	0.91
	UBE2N	0.09	0.53	0.03	0.85
	UBE2G2	0.06	0.71	0.16	0.34
Oxidative stress proteins	SOD1	0.05	0.74	0.09	0.61
	GSTP1	0.11	0.44	0.11	0.52
	GSTA3	0.12	0.43	0.11	0.53
	ESD	0.06	0.69	0.14	0.39
	CAT	0.25	0.09	0.0003	0.99
	SOD2	0.11	0.48	0.08	0.66
	SOD3	0.17	0.25	0.21	0.22

HSP90AA1/AB1: Heat shock protein HSP 90-alpha/beta; HSP70 (HSPA8): Heat shock cognate 71 kDa protein; HSP27 (HSPB1): Heat shock protein beta-1; MAPKAPK5: MAP kinase-activated protein kinase 5; DNAJB1 (HSP40): DnaJ homolog subfamily B member 1; STIP1: Stress-induced-phosphoprotein 1; STUB1: E3 ubiquitin-protein ligase CHIP; UBE2L3: Ubiquitin-conjugating enzyme E2 L3; UBE2N: Ubiquitin-conjugating enzyme E2 N; UBE2G2: Ubiquitin-conjugating enzyme E2 G2; SOD1: Superoxide dismutase; GSTP1: Glutathione S-transferase P; GSTA3: Glutathione S-transferase A3; ESD: Esterase D; CAT: Catalase; SOD2: Superoxide dismutase mitochondrial; and SOD3: Extracellular superoxide dismutase.

## Data Availability

All the data for this study will be made available upon reasonable request to the corresponding author.

## Supplementary Materials

The following supporting information can be downloaded at: https://www.mdpi.com/article/10.3390/biomedicines11072044/s1.
