# Supplementary material for: Oxidative Stress Markers and Heat Shock Proteins in Non-Obese Women with Polycystic Ovary Syndrome Are Not Elevated and Show No Correlation with Vitamin D
Source: Biomedicines. 2023 Jul 20;11(7):2044. doi: 10.3390/biomedicines11072044 (PMC10377564; doi:10.3390/biomedicines11072044)

Supplementary Figure S1. Lack of correlation of oxidative stress proteins with 25(OH)D_3

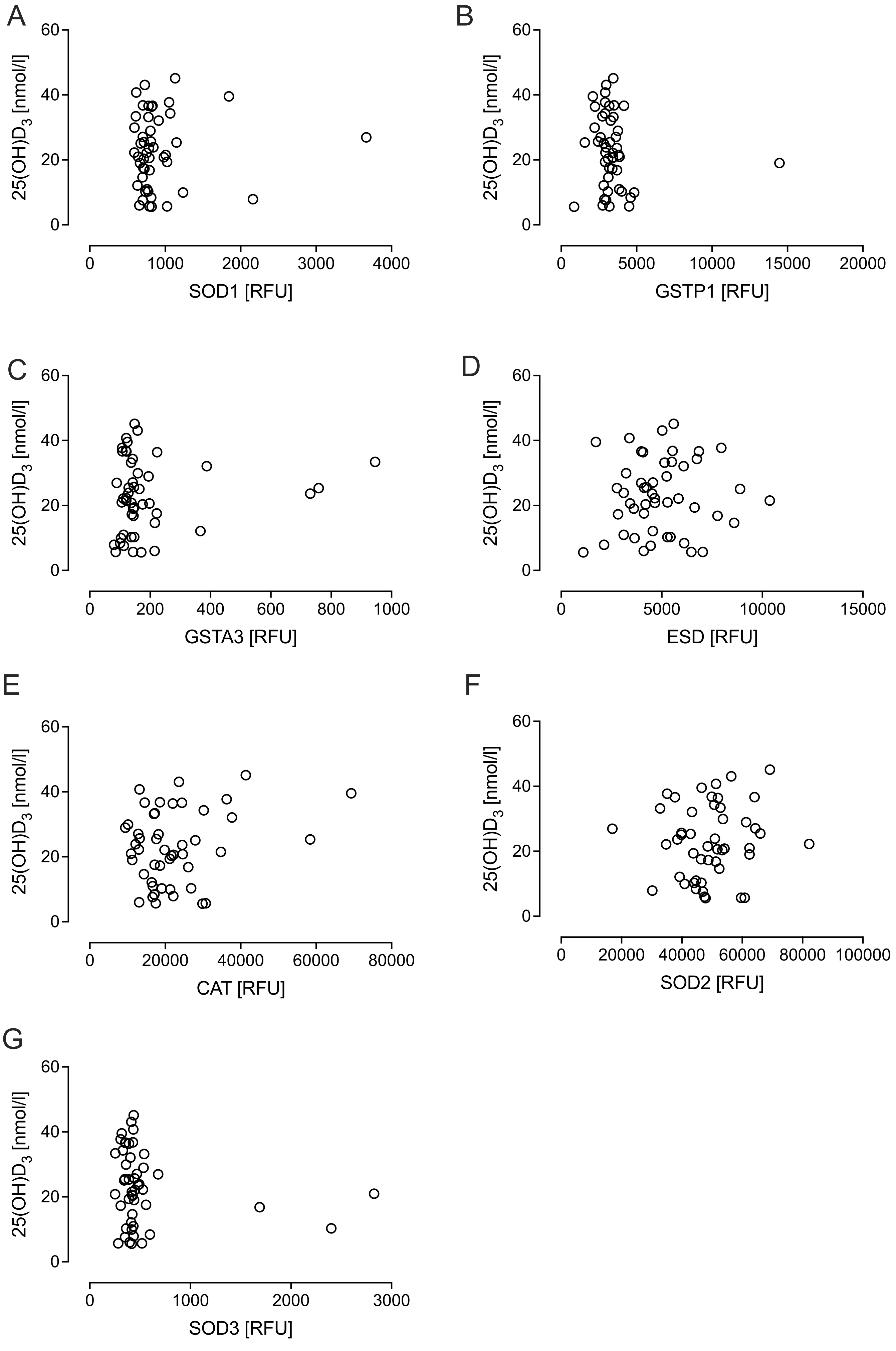

Supplementary Figure S2. Lack of correlation of oxidative stress proteins with $1,25(OH)_2D_3$

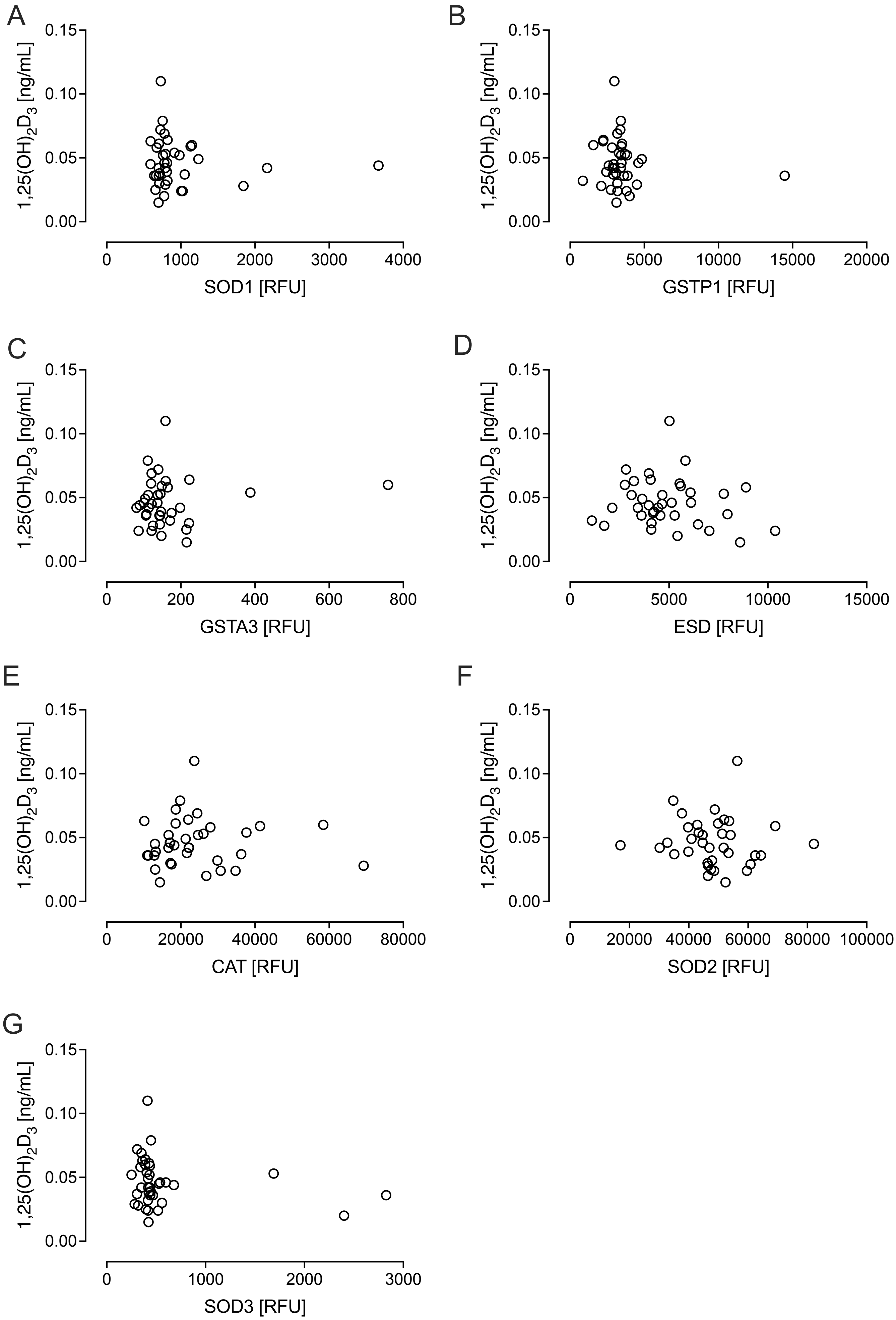

Supplementary Figure S3. Lack of correlation of heat shock proteins with 25(OH)D_3

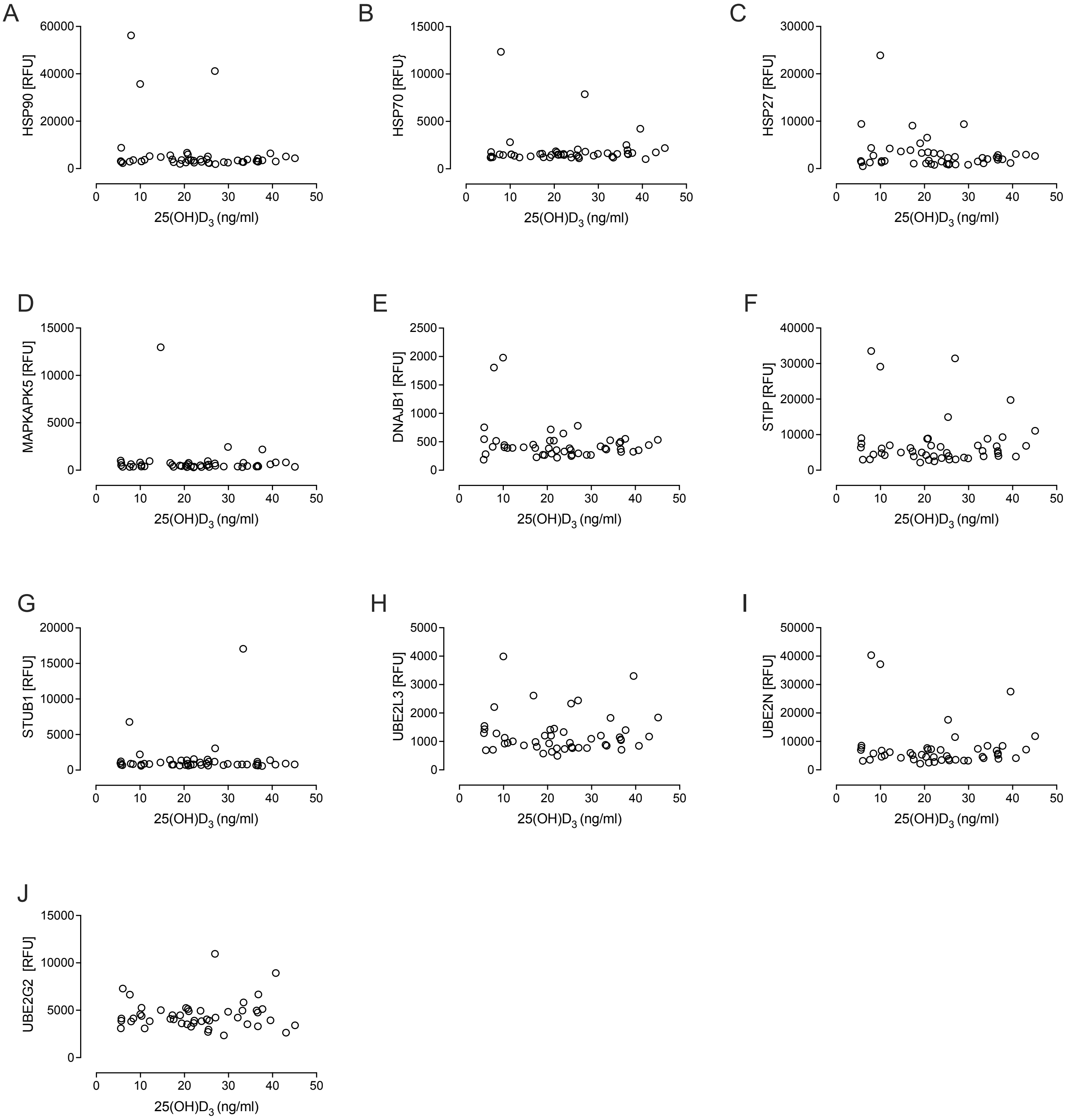

Supplementary Figure S4 .Lack of correlation of heat shock proteins with $1,25(OH)_2D_3$

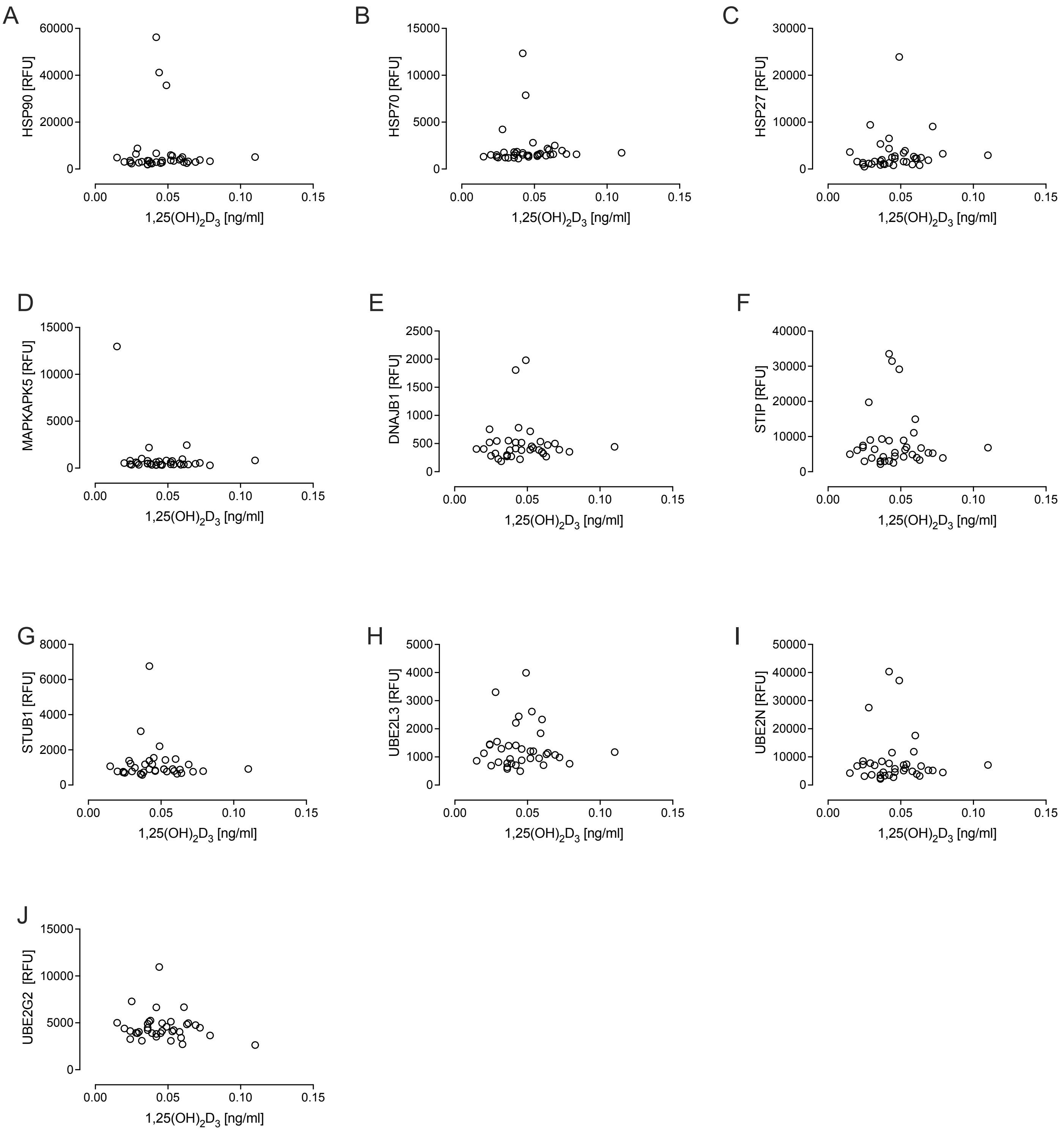

Supplement: Supplementary file 1 [file biomedicines-11-02044-s001.zip › biomedicines-2478034-supplementary.pdf]
